# Impact of *Helicobacter pylori* colonization density and depth on gastritis severity

**DOI:** 10.1186/s12941-024-00666-7

**Published:** 2024-01-12

**Authors:** Jianxiang Peng, Jinliang Xie, Dingwei Liu, Kaijie Yang, Shuang Wu, Dongsheng Liu, Deqiang Huang, Yong Xie

**Affiliations:** 1 Department of Gastroenterology, Digestive Disease Hospital, The First Affiliated Hospital, Jiangxi Medical College, Nanchang University, 17 Yongwaizheng Street, Donghu District, Nanchang, 330006 Jiangxi Province China; 2 Jiangxi Clinical Research Center for Gastroenterology, Nanchang, Jiangxi Province China; 3 Ganzhou People’s Hospital, Ganzhou, Jiangxi Province China; 4 The Second People’s Hospital of Jingdezhen, Jingdezhen, Jiangxi Province China

**Keywords:** *Helicobacter pylori*, Chronic gastritis, Atrophy, Metaplasia

## Abstract

**Background and objectives:**

*Helicobacter pylori* (*H. pylori*) infection is the most common etiology of chronic gastric. *H. pylori* gastritis would gradually evolve into gastric atrophy, intestinal metaplasia, dysplasia and malignant lesions. Herein, this study aimed to investigate the potential impact of *H. pylori* colonization density and depth on the severity of histological parameters of gastritis.

**Methods:**

A prospective monocentric study was conducted from December 2019 to July 2022, enrolling patients with confirmed chronic *H. pylori* infection via histopathological evaluation. *H. pylori* colonization status was detected by immunohistochemical staining, pathological changes of gastric specimens were detected by hematoxylin eosin staining. Epidemiological, endoscopic and histopathological data were collected.

**Results:**

A total of 1120 patients with a mean age of 45.8 years were included. Regardless of the previous history of *H. pylori* eradication treatment, significant correlations were observed between the density and depth of *H. pylori* colonization and the intensity of gastritis activity (all* P* < 0.05). Patients with the lowest level of *H. pylori* colonization density and depth exhibited the highest level of mild activity. In whole participants and anti-*H. pylori* treatment-naive participants, *H. pylori* colonization density and depth were markedly correlated with the severity of chronic gastritis and gastric atrophy (all* P* < 0.05). *H. pylori* colonization density (*P* = 0.001) and depth (*P* = 0.047) were significantly associated with ulcer formation in patients naive to any anti-*H. pylori* treatment. No significant associations were observed between the density and depth of *H. pylori* colonization and other histopathological findings including lymphadenia, lymphoid follicle formation and dysplasia.

**Conclusions:**

As the density and depth of *H. pylori* colonization increased, so did the activity and severity of gastritis, along with an elevated risk of ulcer formation.

**Supplementary Information:**

The online version contains supplementary material available at 10.1186/s12941-024-00666-7.

## Introduction

Gastritis is a prominent pathological condition that may gradually progress to gastric atrophy and cancer, characterized by increased infiltration of the lamina propria with mononuclear leukocytes and polymorphonuclear neutrophils [1]. *Helicobacter pylori* (*H. pylori*) infection plays a major role in the pathogenesis of chronic inflammation in the gastric mucosa [2, 3]. The updated Sydney system is one of the recognized tools for evaluating histological lesions of chronic gastritis and detecting *H. pylori* colonization density [4]. The colonization status of *H. pylori* can also be classified into IV grades and III degrees based on colonization density and depth [5]. Except for chronic gastritis, *H. pylori* is closely related to many malignant and benign upper gastrointestinal diseases including peptic ulcer, mucosa-associated lymphoid tissue (MALT) lymphoma, and gastric cancer [6]. Correa’s Cascade hypothesis suggested that intestinal gastric cancer (Lauren’s type) generally follows the following evolution: normal gastric mucosa → chronic non-atrophic gastritis → chronic atrophic gastritis → intestinal metaplasia → dysplasia → malignant lesions. *H. pylori* infection is considered to be the initiator of Correa’s Cascade hypothesis of gastric mucosal lesions [1]. Therefore, the eradication of *H. pylori* is a crucial strategy to interrupt the carcinogenesis process and prevent the recurrence of peptic ulcer disease [7].

The majority of *H. pylori* strains are located in the gastric pits and mucus gel layer, with only a small proportion colonizing the deeper portions and attaching to gastric cells [8–10]. The pathogenicity of *H. pylori* is influenced by bacterial virulence factors, intensity of bacterial colonization and host genetic factors [11, 12]. However, the correlations between the density and depth of *H. pylori* colonization and the severity of histological parameters of gastritis remains controversial. To the best of our knowledge, no previous study has yet explored these correlations in Chinese population, a region characterized by a high prevalence of *H. pylori* infection and a correspondingly high incidence of gastric cancer.

Hence, the aim of this study was to investigate the potential impact of *H. pylori* colonization density and depth on the severity of histological parameters of gastritis among Chinese patients. Our findings will provide valuable insights for clinicians to devise more effective treatment strategies and improve post-treatment follow-up protocols.

## Materials and methods

This prospective, monocentric study was reviewed and approved by the Medical Ethics Research Committee of the First Affiliated Hospital of Nanchang University (No. 2020–013). All enrolled subjects provided written informed consent prior to participation in the study.

This study carried out in the First Affiliated Hospital of Nanchang University from December 2019 to July 2022. Data sets were collected from medical records of patients who underwent upper endoscopy due to upper gastrointestinal symptoms. Inclusion criteria were patients aged between 18 and 65 years with chronic *H. pylori* infection confirmed by histopathological evaluation. The detailed exclusion criteria were as follows: (1) history of upper gastrointestinal tract surgery; (2) active gastrointestinal bleeding; (3) administration of antibiotics or bismuth agents for 4 weeks prior to upper endoscopy; (4) presence of any serious underlying disease other than dyspepsia; (5) lack of informed consent.

**Fig. 1:**
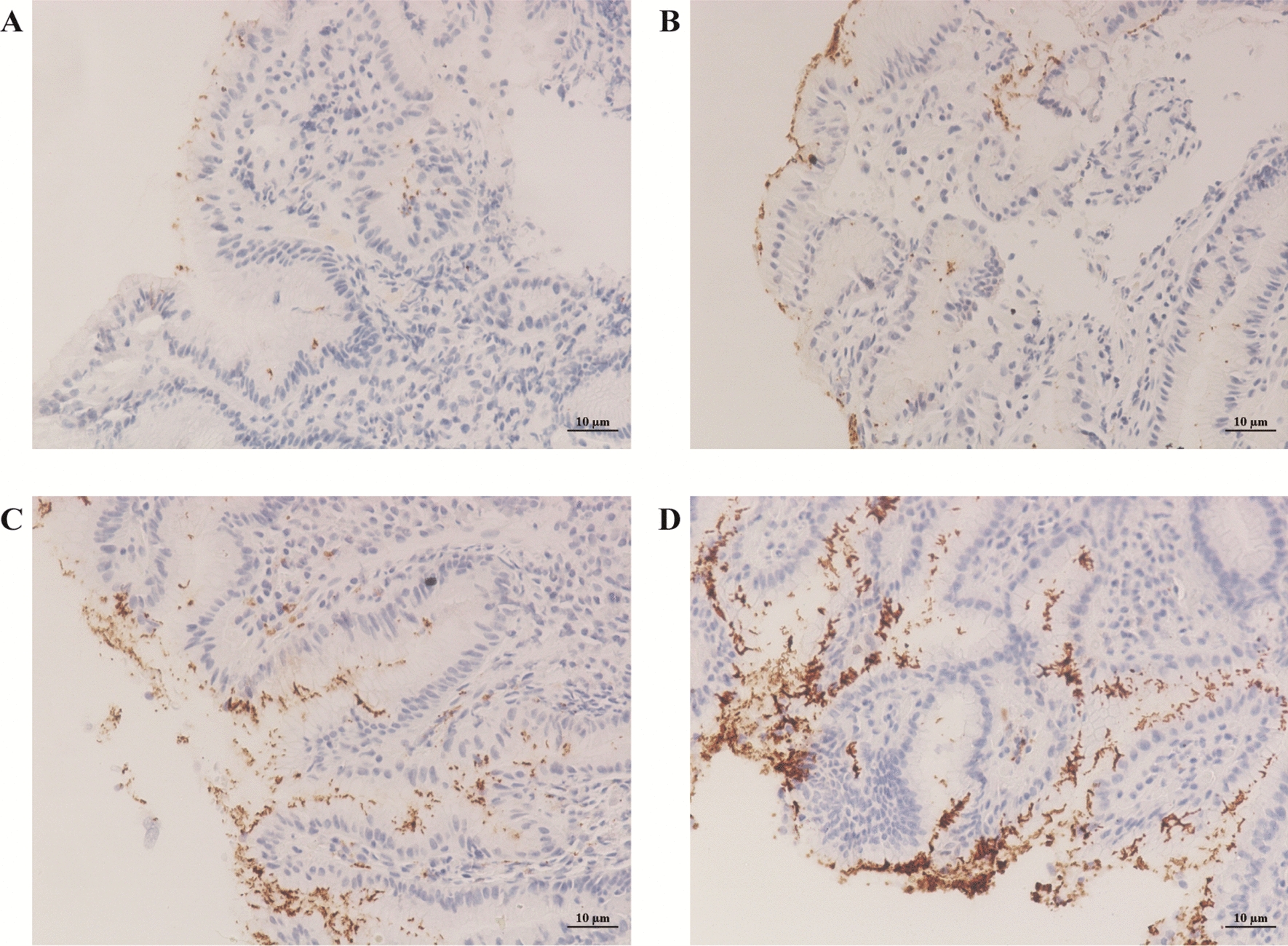
Representative images of different colonization densities and depths of *H. pylori*. **A** grade I and degree I (magnification 200 ×); **B** grade II and degree II (magnification 200 ×); **C** grade III and degree III (magnification 200 ×); **D** grade IV and degree III (magnification 200 ×)

Gastric biopsies were performed by experienced digestive endoscopists. Biopsy specimens were fixed in 10% formalin and then transferred to the pathology department for technical processing and analysis by two senior pathologists. *H. pylori* colonization was detected by immunohistochemical staining [13, 14], pathological changes of gastric specimens were detected by hematoxylin eosin (HE) staining [15]. The status of *H. pylori* colonization can be categorized into IV grades and III degrees based on the colonization density and depth (Fig. 1). The *H. pylori* colonization density was graded as follows: grade I, sporadic presence of bacteria; grade II, bacteria covering less than half of the mucosal surface; grade III, bacteria covering at least half of the mucosal surface; and grade IV, large clusters and deposits of bacteria present. The *H. pylori* colonization depth was classified as follows: degree I, bacteria localized solely on the surface of gastric lumen; degree II, bacteria attached to the upper half of gastric pit; degree III, bacteria deep into the bottom half of gastric pit [5].

The histopathological diagnosis and classification of gastritis was evaluated by visual analogue scale of the updated Sydney system [16] and the pathological diagnostic criteria of chronic gastritis in China [15]. The following parameters were brought into close focus: chronic inflammation, gastritis activity, atrophy and intestinal metaplasia. Each parameter was graded as absent, mild, moderate, or severe. In addition, considering actual situation of pathological diagnosis, atrophy and intestinal metaplasia were both classified as atrophy in this study. When there was a difference in grading between the two pathologists and/or specimens, the highest grade was selected.

All statistical analyses were performed using SPSS 26.0 software. Continuous variables were expressed as mean ± standard deviation. Categorical variables were calculated as frequencies (n) and proportions (%) and compared statistically using the Pearson chi-square test or Fisher’s exact test. When Fisher’s exact test was restricted technically, an asymptotic method was employed. Statistical significance was determined at the bilateral level with *P* < 0.05.

## Results

A total of 1120 patients were included, of which 908 (81.1%) were naive to any anti-*H. pylori* treatment, 212 (18.9%) had received at least one course of anti-*H. pylori* treatment. The average age of enrolled patients was 45.8 years with a standard deviation of 10.68. The mean age for men and women was 45.0 and 46.6 years, respectively. The sex ratio was M/F = 0.91.

Among the 1120 participants, 134 (12.0%) did not exhibit active inflammation, while 352 (31.4%), 598 (53.4%), and 36 (3.2%) experienced mild, moderate, and severe active inflammation, respectively. Additionally, 12 (1.1%) patients had mild chronic gastritis, whereas 990 (88.4%) and 118 (10.5%) suffered from moderate and severe chronic gastritis, respectively.

The majority of participants (77.9%) had superficial erosion, 30.9% had ulceration, 35.8% had gastric atrophy and/or intestinal metaplasia.

In our study, patients with grade I of *H. pylori* colonization density and degree I of colonization depth exhibited the highest level of mild activity. Further, those with grade IV of colonization density and degree III of colonization depth exhibited the highest frequency of severe activity. Regardless of the previous history of *H. pylori* eradication treatment, significant correlations were observed between the density and depth of *H. pylori* colonization and the intensity of gastric mucosa inflammation activity (all P < 0.05). As *H. pylori* colonization density and depth increased, so did the activity level of gastric mucosa inflammation (Table 1).

**Table 1:** Associations between the density and depth of *H. pylori* colonization and activity intensity of inflammation in patients with chronic gastritis (n, %)

Activity intensity	Overall				Treatment-naive patients				Previously treated patients			
	Absent	Mild	Moderate	Severe	Absent	Mild	Moderate	Severe	Absent	Mild	Moderate	Severe
The density of *H. pylori* colonization												
I	20 (33.9)	29 (49.2)	10 (16.9)	0 (0)	15 (31.9)	23 (48.9)	9 (19.1)	0 (0.0)	5 (41.7)	6 (50.0)	1 (8.3)	0 (0.0)
II	38 (20.3)	79 (42.2)	65 (34.8)	5 (2.7)	31 (19.7)	66 (42.0)	55 (35.0)	5 (3.2)	7 (23.3)	13 (43.3)	10 (33.3)	0 (0.0)
III	47 (11.5)	111 (27.1)	237 (57.9)	14 (3.4)	40 (12.0)	88 (26.5)	193 (58.1)	11 (3.3)	7 (9.1)	23 (29.9)	44 (57.1)	3 (3.9)
IV	29 (6.2)	133 (28.6)	286 (61.5)	17 (3.7)	21 (5.6)	107 (28.8)	230 (61.8)	14 (3.8)	8 (8.6)	26 (28.0)	56 (60.2)	3 (3.2)
χ^2^	NA				NA				Na			
*P*	< 0.001				< 0.001				0.002			
The depth of *H. pylori* colonization												
I	21 (36.2)	28 (48.3)	9 (15.5)	0 (0.0)	15 (34.1)	21 (47.7)	8 (18.2)	0 (0.0)	6 (42.9)	7 (50.0)	1 (7.1)	0 (0.0)
II	28 (20.4)	57 (41.6)	48 (35.0)	4 (2.9)	24 (20.4)	48 (41.4)	40 (34.5)	4 (3.4)	4 (19.0)	9 (42.9)	8 (38.1)	0 (0.0)
III	85 (9.2)	267 (28.9)	541 (58.5)	32 (3.5)	68 (9.1)	215 (28.7)	439 (58.7)	26 (3.5)	17 (9.6)	52 (29.4)	102 (57.6)	6 (3.4)
χ^2^	85.152				63.454				Na			
*P*	< 0.001				< 0.001				0.001			

*NA* Asymptotic method

Na: Fisher’s exact test (≥ 20% T < 5 or T > 1)

As shown in Table 2, individuals with grade I of *H. pylori* colonization density and degree I of colonization depth exhibited mostly mild severity of chronic gastritis. Conversely, severe chronic gastritis was reported mostly in people with colonization density and depth of grade IV and degree III, respectively. Overall, there were significant correlations between the density (*P* = 0.026) and depth (*P* = 0.045) of *H. pylori* colonization and the severity of chronic gastritis. These significant correlations were also found in patients naive to any anti-*H. pylori* treatment (for colonization density: *P* = 0.027; for colonization density: *P* = 0.047), but not in those with a history of *H. pylori* eradication.

**Table 2:** Associations between the density and depth of *H. pylori* colonization and level of gastritis severity in patients with chronic gastritis (n, %)

Chronic gastritis severity	Overall			Treatment-naive patients			Previously treated patients		
	Mild	Moderate	Severe	Mild	Moderate	Severe	Mild	Moderate	Severe
The density of *H. pylori* colonization									
I	3 (5.1)	50 (84.7)	6 (10.2)	3 (6.4)	38 (80.9)	6 (12.8)	0 (0.0)	12 (100)	0 (0.0)
II	3 (1.6)	172 (92.0)	12 (6.4)	3 (1.9)	143 (91.1)	11 (7.0)	0 (0.0)	29 (96.7)	1 (3.3)
III	3 (0.7)	365 (89.2)	41 (10.0)	3 (0.9)	293 (88.3)	36 (10.8)	0 (0.0)	72 (93.5)	5 (6.5)
IV	3 (0.6)	403 (86.7)	59 (12.7)	3 (0.8)	317 (85.2)	52 (14.0)	0 (0.0)	86 (92.5)	7 (7.5)
χ^2^	Na			Na			Na		
*P*	0.026			0.027			0.966		
The depth of *H. pylori* colonization									
I	3 (5.2)	51 (87.9)	4 (6.9)	3 (6.8)	37 (84.1)	4 (9.1)	0 (0.0)	14 (100)	0 (0.0)
II	2 (1.5)	124 (90.5)	11 (8.0)	2 (1.7)	104 (89.7)	10 (8.6)	0 (0.0)	20 (95.2)	1 (4.8)
III	7 (0.8)	815 (88.1)	103 (11.1)	7 (0.9)	650 (86.9)	91 (12.2)	0 (0.0)	165 (93.2)	12 (6.8)
χ^2^	Na			Na			Na		
*P*	0.045			0.047			0.851		

Na: Fisher’s exact test (≥ 20% T < 5 or T > 1)

Moreover, in whole participants and anti-*H. pylori* treatment-naive participants, *H. pylori* colonization density and depth were markedly correlated with the severity of gastric atrophy (all* P* < 0.05). In patients with a history of *H. pylori* eradication, atrophy severity was associated with *H. pylori* colonization depth (*P* = 0.015), but not with colonization density (*P* = 0.060). Surprisingly, increasing the density and depth of *H. pylori* colonization decreased the likelihood of detecting atrophy (Table 3).

**Table 3:** Associations between the density and depth of *H. pylori* colonization and level of atrophy severity in patients with chronic gastritis (n, %)

Atrophy severity	Overall				Treatment-naive patients				Previously treated patients			
	Absent	Mild	Moderate	Severe	Absent	Mild	Moderate	Severe	Absent	Mild	Moderate	Severe
The density of *H. pylori* colonization												
I	33 (55.9)	15 (25.4)	6 (10.2)	5 (8.5)	26 (55.3)	12 (25.5)	6 (12.8)	3 (6.4)	7 (58.3)	3 (25.0)	0 (0.0)	2 (16.7)
II	107 (57.2)	58 (31.0)	19 (10.2)	3 (1.6)	96 (61.1)	45 (28.7)	15 (9.6)	1 (0.6)	11 (36.7)	13 (43.3)	4 (13.3)	2 (6.7)
III	271 (66.3)	106 (25.9)	29 (7.1)	3 (0.7)	223 (66.7)	83 (25.0)	24 (7.2)	29 (0.6)	48 (62.3)	23 (29.9)	5 (6.5)	1 (1.3)
IV	308 (66.2)	137 (29.5)	17 (3.7)	3 (0.6)	250 (67.2)	109 (29.3)	11 (3.0)	2 (0.5)	58 (62.4)	28 (30.1)	6 (6.5)	1 (1.1)
χ^2^	NA				NA				Na			
*P*	< 0.001				< 0.001				0.060			
The depth of *H. pylori* colonization												
I	34 (58.6)	13 (22.4)	6 (10.3)	5 (8.5)	26 (59.1)	10 (22.7)	5 (11.4)	3 (6.8)	8 (57.1)	3 (21.4)	1 (7.1)	2 (14.3)
II	82 (59.9)	39 (28.5)	13 (9.5)	3 (2.2)	74 (63.8)	31 (26.7)	10 (8.6)	1 (0.9)	8 (38.1)	8 (38.1)	3 (14.3)	2 (9.5)
III	603 (65.2)	264 (28.5)	52 (5.6)	6 (0.6)	495 (66.2)	208 (27.8)	41 (5.5)	4 (0.5)	108 (61.0)	56 (31.6)	11 (6.2)	2 (1.1)
χ^2^	Na				Na				Na			
*P*	0.001				0.017				0.015			

*NA* Asymptotic method

Na: Fisher’s exact test (≥ 20% T < 5 or T > 1)

In terms of overall participants, there was a statistically significant association between the formation of ulcers and the density of *H. pylori* colonization (*P* = 0.002), while not for the colonization depth (*P* = 0.103). *H. pylori* colonization density (*P* = 0.001) and depth (*P* = 0.047) were significantly associated with ulcer formation among patients naive to any anti-*H. pylori* treatment. That is, by increasing density and depth of *H. pylori* colonization, the likelihood of ulcer formation is increased (Table 4).

**Table 4:** Associations between the density and depth of *H. pylori* colonization and ulcer in patients with chronic gastritis (n, %)

Ulcer	Overall		Treatment-naive patients		Previously treated patients	
	Yes	No	Yes	No	Yes	No
The density of *H. pylori* colonization						
I	12 (20.3)	47 (79.7)	9 (19.1)	38 (80.9)	3 (25.0)	9 (75.0)
II	42 (22.5)	145 (77.5)	37 (23.6)	120 (76.4)	5 (16.7)	25 (83.3)
III	125 (30.6)	284 (69.4)	108 (32.5)	224 (67.5)	17 (22.1)	60 (77.9)
IV	167 (35.9)	298 (64.1)	145 (39.0)	227 (61.0)	22 (23.7)	71 (76.3)
χ^2^	14.819		16.460		0.702	
*P*	0.002		0.001		0.873	
The depth of *H. pylori* colonization						
I	13 (22.4)	45 (77.6)	9 (20.5)	35 (79.5)	4 (28.6)	10 (71.4)
II	35 (25.5)	102 (74.5)	31 (26.7)	85 (73.3)	4 (19.0)	17 (81.0)
III	298 (32.2)	627 (67.8)	259 (34.6)	489 (65.4)	39 (22.0)	138 (78.0)
χ^2^	4.546		6.097		Na	
*P*	0.103		0.047		0.797	

Na: Fisher’s exact test (≥ 20% T < 5 or T > 1)

However, no significant associations were found between the density and depth of *H. pylori* colonization and other histopathological characteristics including lymphadenia, lymphoid follicle formation, glands cystic dilatation, intraepithelial neoplasia, dysplasia, and eosinophil infiltration (Additional file 1: Tables S1–S6).

Afterwards, we discovered a significant correlation between *H. pylori* colonization density and depth in patients with chronic gastritis (*P* < 0.001) (Table 5). As the bacteria colonization density increased, the colonization depth increased as well.

**Table 5:** Correlation between *H. pylori* colonization density and depth in patients with chronic gastritis (n, %)

The depth of *H. pylori* colonization	I	II	III	Total	χ^2^	*P*
The density of *H. pylori* colonization						
I	49 (83.1)	6 (10.2)	4 (6.8)	59 (5.3)	1352.702	< 0.001
II	9 (1.8)	119 (63.6)	59 (31.6)	187 (16.7)		
III	0 (0.0)	10 (2.4)	399 (97.6)	409 (36.5)		
IV	0 (0.0)	2 (0.4)	463 (99.6)	465 (41.5)		
Total	58 (5.2)	137 (12.2)	925 (82.6)	1120 (100.0)		

## Discussion

*H. pylori* infection is a major risk factor for the development of invasive gastric adenocarcinoma, progressing through a multistep process involving active chronic inflammation, non-atrophic chronic gastritis, atrophic gastritis, intestinal metaplasia, and dysplasia [1]. Due to the high prevalence of *H. pylori* infection worldwide, its associated diseases pose a heavy disease burden for human health [17]. Accurate diagnosis and effective eradication of this pathogen not only improve the related gastrointestinal diseases but also reduce the risk of gastric cancer [7, 18]. The purpose of our work is to investigate the impact of *H. pylori* colonization density and depth on the severity of histological parameters of gastritis.

In the present study, nearly 90% of *H. pylori*-infected participants exhibited varying degrees of chronic active gastritis, with moderate activity (53.4%) being the most common. The similar result was found in the study of Souissi et al. [19] conducted in Tunisia.

Our study found significant associations between the density and depth of *H. pylori* colonization and the activity intensity of gastric mucosa inflammation since increasing the density and depth of *H. pylori* colonization increased the activity level of gastritis. Such a result was found in the study conducted in Iran [20] which revealed a significant dose–response association between *H. pylori* colonization density and the intensity of gastritis activity.

The findings of our study also showed significant correlations between the density and depth of *H. pylori* colonization and the severity of chronic gastritis in both overall and treatment-naive patients. While these correlations did not exist in people with a history of *H. pylori* eradication, possibly due to a longer duration of *H. pylori* infection. In previous studies conducted in Tunisia, Iran and Turkey, the relation between the intensity of *H. pylori* infection and the severity of chronic gastritis was all statistically significant [19–21]. However, this relationship was not observed in a study conducted by Park et al. [22] in Korea, nor in a study conducted by Choudhary et al. [23] in Nepal. This discrepancy may be owing to genetic variations, dietary habits, and environmental elements in different study populations.

Gastric atrophy and/or intestinal metaplasia was found in 35.8% of our subjects, which is lower than the percentage reported by Souissi et al. [19] (44.3% for atrophy and 10.3% for intestinal metaplasia) but higher than the percentage reported by Ghasemi et al. [20] (6.7% for atrophy and 12.5% for intestinal metaplasia). The different percentages of atrophy and metaplasia among these studies might be owing to differences in genetic backgrounds, age, dietary habits and the duration of gastritis. Furthermore, we found significant associations between the density and depth of *H. pylori* colonization and the severity of atrophy. The study results of Ghasemi et al. [20] confirmed the findings of our study. Surprisingly, we found that the likelihood of detecting atrophy decreased with increasing density and depth of *H. pylori* colonization, which could be attributed to the fact that the mucosal surface of atrophy and intestinal metaplasia was typically devoid of *H. pylori* colonization [15].

To some extent, *H. pylori* colonization density and depth were positively correlated with ulcer formation in this study. This finding is consistent with the study conducted by Alam et al. [24] in Michigan, which concluded a strong correlation between *H. pylori* colonization density and duodenal ulcer (*P* < 0.001), and a weak association between colonization density and gastric ulcers (*P* = 0.06).

No significant associations were found between the density and depth of *H. pylori* colonization and other histopathological findings, including lymphadenia, lymphoid follicle formation, glands cystic dilatation, intraepithelial neoplasia, dysplasia and eosinophil infiltration. A study conducted in Bangalore indicated that *H. pylori* colonization density was associated with the presence of lymphoid follicles and dysplasia [25]. This disparity might be due to the small number of patients with those histopathological changes in our study population.

Ultimately, we found a robust correlation between the colonization density and depth of *H. pylori* in patients with chronic gastritis, which has not been reported previously. Specifically, as the organism colonization density increases, so does the depth of colonization. When *H. pylori* colonizes gastric mucosa at a high density, the huge bacterial load will produce inoculum effect, which makes *H. pylori* adhere to gastric mucosal cells and form a protective biofilm. Biofilm-forming *H. pylori* is probably prone to survive for an extended period and evade the antimicrobial effects of antibiotics and immune responses of human body, consequently resulting in a persistent inflammatory reaction and tissue damage [26–28]. Studies have shown that CagA( −) bacteria primarily colonized the mucous gel or apical epithelial surface, whereas CagA( +) bacteria colonized the immediate vicinity of epithelial cells or intercellular epithelial spaces [29, 30]. It is reasonable to presume that the bacteria colonizing deeper layers are mainly CagA( +) strains with high pathogenicity. In addition, the bactericidal efficacy of antibiotics on deeply colonized *H. pylori* was diminished due to their inability to penetrate the thick mucus layer, causing persistent inflammatory responses. Therefore, assessing *H. pylori* colonization density and depth may serve as a valuable pre-treatment predictor of eradication therapy effectiveness. We recommend the utilization of more effective regimens, such as bismuth quadruple containing low-resistance antibiotics, to eradicate *H. pylori* in patients with severe bacterial colonization.

The main strengths of our study lie in its prospective design, large sample volume, and investigation of the impact of both *H. pylori* colonization density and depth on the severity of histological parameters of gastritis. Peer studies have mainly focused on the relationship between *H. pylori* colonization density and gastritis severity, but bare of articles have reported the association between *H. pylori* colonization depth and gastritis severity. If our present study was simultaneously conducted in multiple centers, it would possess a higher authority to draw conclusions from our current findings.

## Conclusion

As the density and depth of *H. pylori* colonization increased, so did the activity and severity of gastritis, along with an elevated risk of ulcer formation. Consequently, it can be concluded that *H. pylori* plays a pivotal role in the development and maintenance of chronic active gastritis. These findings highlight the importance of closely monitoring *H. pylori* colonization density and depth, and taking proactive steps to address the infection.

### Supplementary Information

**Additional file 1: Table S1.** Associations between the density and depth of *H. pylori* colonization and lymphadenia in patients with chronic gastritis (n, %). **Table S2.** Associations between the density and depth of *H. pylori* colonization and lymphoid follicle formation in patients with chronic gastritis (n, %). **Table S3.** Associations between the density and depth of *H. pylori* colonization and glands cystic dilatation in patients with chronic gastritis (n, %). **Table S4.** Associations between the density and depth of *H. pylori* colonization and intraepithelial neoplasia in patients with chronic gastritis (n, %). **Table S5.** Associations between the density and depth of *H. pylori* colonization and dysplasia in patients with chronic gastritis (n, %). **Table S6.** Associations between the density and depth of *H. pylori* colonization and eosinophil infiltration in patients with chronic gastritis (n, %).

## Data Availability

The datasets used or analysed during the current study are available from the corresponding author on reasonable request.
