# Supplementary material for: Impact of Helicobacter pylori colonization density and depth on gastritis severity
Source: Ann Clin Microbiol Antimicrob. 2024 Jan 12;23:4. doi: 10.1186/s12941-024-00666-7 (PMC10785438; doi:10.1186/s12941-024-00666-7)
Supplement: Supplementary file 1 — Additional file 1: Table S1. Associations between the density and depth of H. pylori colonization and lymphadenia in patients with chronic gastritis (n, %). Table S2. Associations between the density and depth of H. pylori colonization and lymphoid follicle formation in patients with chronic gastritis (n, %). Table S3. Associations between the density and depth of H. pylori colonization and glands cystic dilatation in patients with chronic gastritis (n, %). Table S4. Associations between the density and depth of H. pylori colonization and intraepithelial neoplasia in patients with chronic gastritis (n, %). Table S5. Associations between the density and depth of H. pylori colonization and dysplasia in patients with chronic gastritis (n, %). Table S6. Associations between the density and depth of H. pylori colonization and eosinophil infiltration in patients with chronic gastritis (n, %). [file 12941_2024_666_MOESM1_ESM.doc]

**TABLE S1 Associations between the density and depth of *H. pylori* colonization and lymphadenia in patients with chronic gastritis (n, %)**

| Lymphadenia | Overall |  |  | Treatment-naive patients | |  | Previously treated patients | |
| --- | --- | --- | --- | --- | --- | --- | --- | --- |
|  | Yes | No |  | Yes | No |  | Yes | No |
| The density of *H. pylori* colonization | | |  |  |  |  |  |  |
| I | 17 (28.8) | 42 (71.2) |  | 13 (27.7) | 34 (72.3) |  | 4 (33.3) | 8 (66.7) |
| II | 53 (28.3) | 134 (71.7) |  | 47 (29.9) | 110 (70.1) |  | 6 (20.0) | 24 (80.0) |
| III | 87 (21.3) | 322 (78.7) |  | 74 (22.3) | 258 (77.7) |  | 13 (16.9) | 64 (83.1) |
| IV | 108 (23.2) | 357 (76.8) |  | 91 (24.5) | 281 (75.5) |  | 17 (18.3) | 76 (81.7) |
| χ^2^ | 4.478 |  |  | 3.574 |  |  | 1.885 |  |
| *P* | 0.214 |  |  | 0.311 |  |  | 0.597 |  |
| The depth of *H. pylori* colonization | | |  |  |  |  |  |  |
| I | 17 (29.3) | 41 (70.7) |  | 12 (27.3) | 32 (72.7) |  | 5 (35.7) | 9 (64.3) |
| II | 38 (27.7) | 99 (72.3) |  | 35 (30.2) | 81 (69.8) |  | 3 (14.3) | 18 (85.7) |
| III | 210 (22.7) | 715 (77.3) |  | 178 (23.8) | 570 (76.2) |  | 32 (18.1) | 145 (81.9) |
| χ^2^ | 2.755 |  |  | 2.344 |  |  | Na |  |
| *P* | 0.252 |  |  | 0.310 |  |  | 0.279 |  |

Na: Fisher’s exact test ( ≥ 20% T < 5 or T > 1)

**TABLE S2 Associations between the density and depth of *H. pylori* colonization and lymphoid follicle formation in patients with chronic gastritis (n, %)**

| Lymphoid follicle formation | Overall |  |  | Treatment-naive patients | |  | Previously treated patients | |
| --- | --- | --- | --- | --- | --- | --- | --- | --- |
|  | Yes | No |  | Yes | No |  | Yes | No |
| The density of *H. pylori* colonization | | |  |  |  |  |  |  |
| I | 4 (6.8) | 55 (93.2) |  | 4 (8.5) | 42 (91.5) |  | 0 (0.0) | 12 (100) |
| II | 8 (4.3) | 179 (95.7) |  | 8 (5.1) | 149 (94.9) |  | 0 (0.0) | 30 (100) |
| III | 19 (4.6) | 390 (95.4) |  | 17 (5.1) | 315 (94.9) |  | 2 (2.6) | 75 (97.4) |
| IV | 22 (4.7) | 443 (95.3) |  | 21 (5.6) | 351 (94.4) |  | 1 (1.1) | 92 (98.6) |
| χ^2^ | 0.641 |  |  | 0.975 |  |  | Na |  |
| *P* | 0.887 |  |  | 0.807 |  |  | 0.790 |  |
| The depth of *H. pylori* colonization | | |  |  |  |  |  |  |
| I | 4 (6.9) | 54 (93.1) |  | 4 (9.1) | 40 (90.9) |  | 0 (0.0) | 14 (100) |
| II | 7 (5.1) | 130 (94.9) |  | 7 (6.0) | 109 (94.0) |  | 0 (0.0) | 21 (100) |
| III | 42 (4.5) | 883 (95.9) |  | 39 (5.2) | 709 (94.8) |  | 3 (1.7) | 174 (98.3) |
| χ^2^ | 0.721 |  |  | 1.272 |  |  | Na |  |
| *P* | 0.697 |  |  | 0.529 |  |  | 1.000 |  |

Na: Fisher’s exact test ( ≥ 20% T < 5 or T > 1)

**TABLE S3 Associations between the density and depth of *H. pylori* colonization and** **glands cystic dilatation in patients with chronic gastritis (n, %)**

| Glands cystic dilatation | Overall |  |  | Treatment-naive patients | |  | Previously treated patients | |
| --- | --- | --- | --- | --- | --- | --- | --- | --- |
|  | Yes | No |  | Yes | No |  | Yes | No |
| The density of *H. pylori* colonization | |  |  |  |  |  |  |  |
| I | 2 (3.4) | 57 (96.6) |  | 2 (4.3) | 45 (95.7) |  | 0 (0.0) | 12 (100) |
| II | 3 (1.6) | 184 (98.4) |  | 1 (0.6) | 156 (99.4) |  | 2 (6.7) | 28 (93.3) |
| III | 5 (1.2) | 404 (98.8) |  | 4 (1.2) | 328 (98.8) |  | 1 (1.3) | 76 (98.7) |
| IV | 4 (0.9) | 461 (99.1) |  | 3 (0.8) | 369 (99.2) |  | 1 (1.1) | 92 (98.9) |
| χ^2^ | Na |  |  | Na |  |  | Na |  |
| *P* | 0.327 |  |  | 0.206 |  |  | 0.285 |  |
| The depth of *H. pylori* colonization | |  |  |  |  |  |  |  |
| I | 1 (1.7) | 57 (98.3) |  | 1 (2.3) | 43 (97.7) |  | 0 (0.0) | 14 (100) |
| II | 3 (2.2) | 134 (97.8) |  | 2 (1.7) | 114 (98.3) |  | 1 (4.8) | 20 (95.2) |
| III | 10 (1.1) | 915 (98.9) |  | 7 (0.9) | 741 (99.1) |  | 3 (1.7) | 174 (98.3) |
| χ^2^ | Na |  |  | Na |  |  | Na |  |
| *P* | 0.290 |  |  | 0.273 |  |  | 0.517 |  |

Na: Fisher’s exact test ( ≥ 20% T < 5 or T > 1)

**TABLE S4 Associations between the density and depth of *H. pylori* colonization and** **intraepithelial neoplasia in patients with chronic gastritis (n, %)**

| Intraepithelial neoplasia | Overall |  |  | Treatment-naive patients | |  | Previously treated patients | |
| --- | --- | --- | --- | --- | --- | --- | --- | --- |
|  | Yes | No |  | Yes | No |  | Yes | No |
| The density of *H. pylori* colonization | |  |  |  |  |  |  |  |
| I | 1 (1.7) | 58 (98.3) |  | 1 (2.1) | 46 (97.9) |  | 0 (0.0) | 12 (100) |
| II | 3 (1.6) | 184 (98.4) |  | 3 (1.9) | 154 (98.1) |  | 0 (0.0) | 30 (100) |
| III | 4 (1.0) | 405 (99.0) |  | 4 (1.2) | 328 (98.8) |  | 0 (0.0) | 77 (100) |
| IV | 3 (0.6) | 462 (99.4) |  | 3 (0.8) | 369 (99.2) |  | 0 (0.0) | 93 (100) |
| χ^2^ | Na |  |  | Na |  |  | - |  |
| *P* | 0.464 |  |  | 0.509 |  |  | - |  |
| The depth of *H. pylori* colonization | |  |  |  |  |  |  |  |
| I | 0 (0.0) | 58 (100) |  | 0 (0.0) | 44 (100) |  | 0 (0.0) | 14 (100) |
| II | 2 (1.5) | 135 (98.5) |  | 2 (1.7) | 114 (98.3) |  | 0 (0.0) | 21 (100) |
| III | 9 (1.0) | 916 (99.0) |  | 9 (1.2) | 739 (98.8) |  | 0 (0.0) | 177 (100) |
| χ^2^ | Na |  |  | Na |  |  | - |  |
| *P* | 0.801 |  |  | 0.798 |  |  | - |  |

Na: Fisher’s exact test ( ≥ 20% T < 5 or T > 1)

**TABLE S5 Associations between the density and depth of *H. pylori* colonization and** **dysplasia in patients with chronic gastritis (n, %)**

| Dysplasia | Overall |  |  | Treatment-naive patients | |  | Previously treated patients | |
| --- | --- | --- | --- | --- | --- | --- | --- | --- |
|  | Yes | No |  | Yes | No |  | Yes | No |
| The density of *H. pylori* colonization | |  |  |  |  |  |  |  |
| I | 1 (1.7) | 58 (98.3) |  | 1 (2.1) | 46 (97.9) |  | 0 (0.0) | 12 (100) |
| II | 1 (0.5) | 186 (99.5) |  | 1 (0.6) | 156 (99.4) |  | 0 (0.0) | 30 (100) |
| III | 1 (0.2) | 408 (99.8) |  | 1 (0.3) | 331 (99.7) |  | 0 (0.0) | 77 (100) |
| IV | 6 (1.3) | 459 (98.7) |  | 4 (1.1) | 368 (98.9) |  | 2 (2.2) | 91 (97.8) |
| χ^2^ | Na |  |  | Na |  |  | Na |  |
| *P* | 0.196 |  |  | 0.296 |  |  | 0.680 |  |
| The depth of *H. pylori* colonization | |  |  |  |  |  |  |  |
| I | 1 (1.7) | 57 (98.3) |  | 1 (2.3) | 43 (97.7) |  | 0 (0.0) | 14 (100) |
| II | 0 (0.0) | 137 (100) |  | 0 (0.0) | 116 (100) |  | 0 (0.0) | 21 (100) |
| III | 8 (0.9) | 917 (99.1) |  | 6 (0.8) | 742 (99.2) |  | 2 (1.1) | 176 (98.9) |
| χ^2^ | Na |  |  | Na |  |  | Na |  |
| *P* | 0.322 |  |  | 0.333 |  |  | 1.000 |  |

Na: Fisher’s exact test ( ≥ 20% T < 5 or T > 1)

**TABLE S6 Associations between the density and depth of *H. pylori* colonization and** **eosinophil infiltration in patients with chronic gastritis (n, %)**

| Eosinophil infiltration | Overall |  |  | Treatment-naive patients | |  | Previously treated patients | |
| --- | --- | --- | --- | --- | --- | --- | --- | --- |
|  | Yes | No |  | Yes | No |  | Yes | No |
| The density of *H. pylori* colonization | |  |  |  |  |  |  |  |
| I | 0 (0.0) | 59 (100) |  | 0 (0.0) | 47 (100) |  | 0 (0.0) | 12 (100) |
| II | 0 (0.0) | 187 (100) |  | 0 (0.0) | 157 (100) |  | 0 (0.0) | 30 (100) |
| III | 1 (0.2) | 408 (99.8) |  | 1 (0.3) | 331 (99.7) |  | 0 (0.0) | 77 (100) |
| IV | 5 (1.1) | 460 (98.9) |  | 4 (1.1) | 368 (98.9) |  | 1 (1.1) | 92 (98.9) |
| χ^2^ | Na |  |  | Na |  |  | Na |  |
| *P* | 0.323 |  |  | 0.497 |  |  | 1.000 |  |
| The depth of *H. pylori* colonization | |  |  |  |  |  |  |  |
| I | 0 (0.0) | 58 (100) |  | 0 (0.0) | 44 (100) |  | 0 (0.0) | 14 (100) |
| II | 0 (0.0) | 137 (100) |  | 0 (0.0) | 116 (100) |  | 0 (0.0) | 21 (100) |
| III | 6 (0.6) | 919 (99.4) |  | 5 (0.7) | 743 (99.3) |  | 1 (0.6) | 176 (99.4) |
| χ^2^ | Na |  |  | Na |  |  | Na |  |
| *P* | 1.000 |  |  | 1.000 |  |  | 1.000 |  |

Na: Fisher’s exact test ( ≥ 20% T < 5 or T > 1)
